# Phosphate solubilizers enhance NPK fertilizer use efficiency in rice and legume cultivation

**DOI:** 10.1007/s13205-011-0028-2

**Published:** 2011-10-21

**Authors:** I. Duarah, M. Deka, N. Saikia, H. P. Deka Boruah

**Affiliations:** 1 Biotechnology Division, NEIST (CSIR), Jorhat, 785006 Assam India; 2 Department of Biotechnology, Guwahati University, Gauhati, Assam India

**Keywords:** Fertilizer, Phosphate solubilizing bacteria, *Oryza sativa*, *Vigna unguiculata*, Germination index, Plant growth

## Abstract

It has been reported that phosphate solubilizing bacteria (PSB) are the most promising bacteria among the plant growth promoting rhizobacteria (PGPR); which may be used as biofertilizers for plant growth and nutrient use efficiency. Moreover, these soil micro-organisms play a significant role in regulating the dynamics of organic matter decomposition and the availability of plant nutrients such as nitrogen (N), phosphorus (P), potassium (K) and other nutrients. Through this study, the management of nutrient use efficiency by the application of PSB was targeted in order to make the applied nutrients more available to the plants in the rice (*Oryza sativa*) and yardlong bean (*Vigna unguiculata*) cultivation. Results have shown that the treatments with PSB alone or in the form of consortia of compatible strains with or without the external application of chemical NPK gave more germination index (G. I.) from 2.5 to 5 in rice and 2.7 to 4.8 in bean seeds. They also showed a higher growth in both shoot and root length and a higher biomass as compared to the control. This gives us an idea about the potentiality of these PSB strains and their application in rice and yardlong bean cultivation to get a better harvest index. Their use will also possibly reduce the nutrient runoff or leaching and increase in the use efficiency of the applied fertilizers. Thus, we can conclude that the NPK uptake and management can be improved by the use of PSB in rice and yardlong bean cultivation, and their application may be much more beneficial in the agricultural field.

## Introduction

The application of mineral fertilizers is the most advantageous and the fastest way to increase crop yields. In the last few decades the rate of nitrogen (N), phosphorous (P) and potassium (K) or NPK fertilizer application has tremendously increased in crop production (Adesmoye and Kloepper 2009). The excessive use of synthetic agrochemicals in crop production and in soil fertility management causes residue toxicity and environmental pollution (Tilman et al*.*^2002^; Adesmoye and Kloepper 2009). This is due to low use efficiency of externally applied fertilizers by the plants, long-term application, leaching, and evaporation to atmosphere (Tilman 1998; Gyaneshwar et al*.*^2002^; Kennedy et al. 2004). Therefore, the reduced use of synthetic agrochemicals in crop production and to maintain soil fertility by alternative means is the subject of investigation. The challenge is to continue sustainable agricultural crop production through minimization of harmful effect of fertilization.

Among the different alternatives, researchers hypothesized that plant growth promoting rhizobacteria (PGPR) could be a substitute to these (Kloepper and Schroth 1978; Glick 1995; Deka Boruah and Dileep Kumar 2002; Glick et al. 2007). Initially, though the application of PGPR was concentrated on biological control (Suslow 1982; Glick 1995), the use of PGPR is also being investigated for nutrient use efficiency in crop plants (Adesmoye and Kloepper 2009; Hariprasad and Niranjana 2009). They can be employed for efficient use of externally applied fertilizers (Vessey 2003; Adesmoye and Kloepper 2009). Among the different PGPR, phosphate solubilizing bacteria (PSB) on plant growth and development, their application in nutrient management and fertilizer use efficiency were earlier reported by several researchers (Rodriguez and Fraga 1999; Richardson 2001; Peix et al. 2001; Vessey 2003; Poonguzhali et al. 2007; Adesmoye and Kloepper 2009; Hariprasad and Niranjana 2009; Kim et al. 2009; Hong et al*.*^2010^). The use of phosphate solubilizers in association with a host plant results in stimulation of growth of their host plants. Plant growth promotion may be achieved directly by the ability of the bacteria to fix nitrogen, sequester iron, facilitate phosphorus uptake, and produce phytohormones that triggers responses in a growing plant exerting multiple effects on plant growth and soil fertility improvement (Rodriguez and Fraga 1999; Peix et al. 2001; Glick et al. 2007; Hariprasad and Niranjana 2009; Kim et al. 2009). The PGPR are also reported to be involved in seed germination and somehow in the induction of α-amylase, which helps to provide energy for the growth of roots and shoots (Beck and Ziegler 1989; Kaneko et al. 2002). The induction of α-amylase also is a lead to the biosynthesis of phytohormones such as gibberellins that stimulates the plant growth (Fincher 1989; Kaneko et al. 2002).

Considering the authenticity of the aforesaid works, it can be put forward that if we introduce PSB in crop field with or without the application of chemical fertilizer, the doses of agrochemicals may be reduced as well as the crop productivity can be increased. Present investigation was carried out to study the assessment of the role of the PSB in NPK management and their effect in reduction of NPK use in the crop production as well as on germination and growth of Rice (*Oryza sativa*) and yardlong bean (*Vigna unguiculata*). Rice (*Oryza sativa*) is the most important grain with regards to human nutrition and caloric intake, providing more than one-fifth of the calories consumed worldwide by the human species (Paul et al. 2000). The yardlong bean (*Vigna unguiculata*), is a good source of protein, vitamin A, thiamin, riboflavin, iron, phosphorus, and potassium, and a very good source for vitamin C, folate, magnesium, and manganese (National research council 2006). The production of these two crops needs external application of fertilizers. Considering the worldwide importance of these two crops, the application of phosphate solubilizers in rice and yardlong bean cultivation was considered to study the efficiency of PGPR in nutrient management. Though various factors determine the efficiency of PGPR in nutrient management, their application is increasing because of its ecofriendly and cost effective nature. In this study, the effort was given for the management of nutrient use efficiency through the application of PSB in order to make the applied nutrients more available to the plant.

## Materials and methods

### Strains

Seven numbers of PSB were taken for the experiment from the Biotechnology division, NEIST, Jorhat, which were selected on the basis of their higher phosphate solubilizing efficiency (more than 50% p-solubilizing efficiency) on Pikovskaya medium (Pikovskaya 1948) and their positive response to the production of siderophore, plant growth regulators indole-acetic acid (IAA) and gibberellic acid (GA), etc. Details of the strains are given in Table 1.

**Table 1:** Biochemical characters and the accession nos. of the strains under the study

Strains	Name	Accession no.	Siderophore	IAA	GA
TP06	*Staphylococcus epidermidis*	FJ887885	+	+	+
TP08	*Erwinia tasmaniensis*	FJ887889	+	+	+
TP13	*Pseudomonas aeruginosa*	Not submitted	+	+	+
TP14	*Pseudomonas aeruginosa*	–	+	+	+
TP16	*Pseudomonas aeruginosa*	–	+	+	+
TP27(7)	*Bacillus subtilis*	FJ887881	+	+	+
TP373	*Pseudomonas aeruginosa*	FJ887882	+	+	+

### Experimental soil

The soil used for the experiment was taken from the North East Institute of Science and Technology, Jorhat (NEIST) campus from a depth of 0–10 cm and laboratory analysis was done for physicochemical and biological characteristics. The soil pH was determined in 1:2.5 soil:water suspensions using an automatic glass electrode pH meter (Systronic model-361). The soil moisture content was determined after drying the soil samples at 105 °C until a constant weight was obtained. The percentage of organic C was determined by Walkley and Black (1934) while phosphorus was evaluated by phosphomolybdic acid methods (Trivedy et al*.*^1998^). Total nitrogen was estimated through Kjeldahl digestion.

Soil enzyme activity phosphatase was determined using the method described by Tabatabai and Bremner (1969). The optical density was recorded at 430 nm using an Analyticzena (Specord 210) spectrophotometer to determine how much *p*-nitrophenol was released and was quantified from standard curve of *p*-nirophenol. The 2,3,5-triphenyltetrazolium chloride (TTC) reduction technique (Casida 1964) was used to measure the soil dehydrogenase activity. The total dehydrogenase activity was calculated from standard curve of sodium hydrosulphide and total activity was expressed as mg INTF g^−1^ dry soil h^−1^. The urease activity was measured using the method described by Mc Garity and Myers (1967). The amount of NH_4_-N released by the urease enzyme was calculated using a reference-calibrated curve of NH_4_OH and expressed as NH_4_-N mg g^−1^ of dry soil h^−1^.

### Experimental setup

Plant growth promoting activity and NPK use efficiency of PSB on rice (*Oryza sativa)* and yardlong bean (*Vigna unguiculata*) was observed on filter paper in the laboratory condition and in earthen pots in the field by the methods of Deka Boruah and Dileep Kumar (2002). First, the earthen pots (6 cm × 12 cm × 14 cm) were filled with fertile soil with or without NPK (2:1:1) in 4 and 8 g pot^−1^; i.e., 50 and 100% NPK of the recommended dose (the application dose for 100% and 50% was 40:20:20 and 20:20:10 g NPK per 50 kg of soil). The sources for NPK were urea, superphosphate and murate of potash, respectively. For the study on filter paper solutions of NPK were made at 50 and 100% concentrations and applied on filter paper. The treatments made for the entire period of investigation for both the plants were—treatment 1 (T1): garden soil; treatment 2 (T2): garden soil + NPK (100%); treatment 3 (T3): garden soil + NPK (50%), treatment 4 (T4): garden soil + TP08; treatment 5 (T5): garden soil + TP16; treatment 6 (T6): garden soil + TP373; treatment 7 (T7): garden soil + consortia (TP06 + TP13 + TP14 + TP 27(7)); treatment 8 (T8): garden soil + TP08 + 50% NPK; treatment 9 (T9): garden soil + TP16 + 50% NPK; treatment 10 (T10): garden soil + TP373 + 50% NPK; treatment 11 (T11): garden soil + consortia [TP06 + TP13 + TP14 + TP 27(7)] + 50% NPK.

Surface sterilization and seed bacterization of both rice and yardlong bean seeds was done according to the method of Deka Boruah and Dileep Kumar (2002). For the rice seeds, according to Colmer (2003); the flask containing the seeds was covered with carbon paper in order to provide a dark condition for the emergence of coleoptiles (S1 stage) and then surface sterilized. For seed bacterization, surface sterilized seeds were then imbibed in a bacterial cell suspension having the colony forming unit (cfu) 1.2 × 10^7^ for 4 h in a reciprocal shaker adjusted to 60 rpm. After 4 h of imbibing, the seeds were removed and dried under laminar air flow. The seeds were then transferred to a filter paper and earthen pots; allowed to grow for 15 days. Seeds (without seed bacterization) treated with PNS (Plant nutrient solution) alone was considered as control. The seeds on filter papers were then allowed to germinate under controlled condition at 25 °C in plant growth chamber with 10 h photoperiod, while the earthen pots were transferred to the greenhouse. The effect of PSB on root length, shoot lengths, total root and shoot biomass was recorded after 15th day. All the treatments were repeated at least twice with six replications each.

### Availability and uptake of NPK

Availability of NPK was analyzed in the soil of each treatment at initial stage and after 30 days of treatment. Effect of PSB on nutrient uptake of rice and yardlong bean was analyzed in the leaves of 30 days old seedlings. Total N was analyzed by Kjeldahl digestion methods, total P in plant samples was estimated by ammonium-molebdate method in acid digestion methods; whereas K was analyzed by flame photometrically.

### Speed of germination (G. I. index)

Number of seedlings emerging daily were counted from the day of planting the seeds in the medium till the time germination (up to 5th day) is complete. Thereafter, a germination index (G. I.) is computed by using the following formula (Elliott 2001*)*:where, *n* = number of seedlings emerging on day ‘d’; *d* = day after planting.

### Shoot length, root length and biomass

The root length, shoot lengths, total root and shoot biomass of both the yardlong bean and rice seedlings were recorded after 15th day of treatment; till the V3 stage (collar formation of leaf 3 on main stem) in case of rice. All the treatments were repeated at least five times with five replications each.

### Amylase activity

The amylase activity in seeds at the post-germination stage and on the leaves of seedlings (5th day of germination) was assayed by the method of Thiammaiah (2004). Extraction of *α*-amylase was done in 10 mM ice cold CaCl_2_ solution for 3 h at room temperature. For free β-amylase, 1 gm acetone defatted material was crushed with 66 mM phosphate buffer (pH 7.0) buffer at 4 °C and supernatant was taken for β-amylase assay. For the enzyme assay, 1.0 mL properly diluted enzyme was mixed with 1% starch solution and then incubated for 15 min at 27 °C. Then the reaction was stopped by adding di-nitro salicylic acid solution and incubated in a boiling water bath for 5 min and 1.0 mL of sodium potassium tartarate was added, the absorbance was recorded under 560 nm against the blank. The amylase activity was calculated by using a standard curve of maltose and expressed as μg of maltose released per gm of fresh weight of the material in 1 h.

### Statistical analysis

Statistical analysis were carried out through one-way analysis of variance (ANOVA) to see the significance difference and the mean of treatments were compared according to Tukey’s test at *p* ≤ 0.001, *p* ≤ 0.01 and *p* ≤ 0.05. All the analysis was done in Graph pad Prism5 programme.

## Results and discussions

### The experimental soil

The pH of the experimental soil was moderately acidic (5.2–5.9) in all the soil samples before and after treatments. However, a decrease in pH from 5.9 to 5.6 was found in 100% NPK treated soil for rice and 5.9 in yard long bean. The moisture content and the carbon content of the soil were estimated at 25.9 and 0.3–1.2%, respectively. The respective experimental soil pH is suitable for growing both yardlong bean and rice. This is supported by the earlier studies as yardlong bean prefer a light, well-drained soil with a pH of 5.5–6.8; while the rice cultivation needs pH of 5.0–6.0 (National research council 2006; Paul et al. 2000).

The dehydrogenase activity of soil in case of both rice and bean cultivation was found to be decreased with the amendment of NPK fertilizer, which is evident in 100% NPK (1.51−0.77 mg g^−1^ h^−1^) and 50% NPK (2.10–0.83 mg g^−1^ h^−1^) after the 30th day of the experiment (Table 2). In contrast to this, a significant increase of dehydrogenase activity (*p* ≤ 0.05) was found in PSB treated (without NPK treatment) soil over the control. Whereas no such increase was found in case of soil treated with both NPK and PSB. Dehydrogenase activity is commonly used as an indicator for biological activity, i.e., it can be used to indicate the total microbial population. The decrease in dehydrogenase activity in NPK amended soil is contributed by its toxic effect on total soil microbes. This explanation can also be co-related with the work of Tilak et al. (2005), as he noted that the intensive use of chemical fertilizer results in increased soil salinity, which leads to deterioration of soil health. Dehydrogenase enzyme exhibits lower activity at higher doses of pesticides, which was reported by Baruah and Mishra (1986). However, the decrease in dehydrogenase activity may also be contributed by the fact that indicator TTC (triphenyltetrazolium chloride) yield less formazan for the interference by the NPK with the electron donors (Casida 1977).

**Table 2:** Comparison of microbial activity in the experimental soil at the initial stage and after 30th day of the experiment on *Oryza sativa* and *Vigna unguiculata*

Treatments	Dehydrogenase activity (mg g^−1^ h^−1^)			Urease activity (mg g^−1^ h^−1^)			Phosphatase activity (mg g^−1^ h^−1^)		
	At 0 day	At 30th day		At 0 day	At 30th day		At 0 day	At 30th day	
		*Oryza sativa*	*Vigna unguiculata*		*Oryza sativa*	*Vigna unguiculata*		*Oryza sativa*	*Vigna unguiculata*
T1	1.43 ± 0.002ab	1.45 ± 0.007ab	1.59 ± 0.009ab	0.38 ± 0.007a	1.0 ± 0.011b	1.1 ± 0.008c	1.04 ± 0.005c	2.09 ± 0.007c	2.09 ± 0.008c
T2	1.51 ± 0.007a	0.77 ± 0.008cd	0.62 ± 0.011c	0.47 ± 0.008a	1.1 ± 0.012b	1.2 ± 0.009c	1.07 ± 0.007c	2.15 ± 0.008bc	2.16 ± 0.008b
T3	2.10 ± 0.012a	0.83 ± 0.011c	0.62 ± 0.012c	0.49 ± 0.008a	1.2 ± 0.011b	1.9 ± 0.006bc	1.05 ± 0.007c	2.16 ± 0.003bc	2.18 ± 0.007b
T4	2.00 ± 0.014a	2.01 ± 0.012a	2.21 ± 0.012a	0.32 ± 0.005a	2.4 ± 0.008a	3.4 ± 0.012a	1.14 ± 0.011b	2.21 ± 0.005b	2.19 ± 0.008a
T5	1.29 ± 0.008ab	2.23 ± 0.016a	1.90 ± 0.005a	0.39 ± 0.005a	2.1 ± 0.009a	2.4 ± 0.011b	1.35 ± 0.015a	2.23 ± 0.012b	2.21 ± 0.007a
T6	1.43 ± 0.009ab	2.34 ± 0.006a	2.03 ± 0.008a	0.45 ± 0.006a	2.3 ± 0.005a	2.4 ± 0.007b	1.14 ± 0.005b	2.19 ± 0.003b	2.19 ± 0.008a
T7	1.52 ± 0.004a	1.46 ± 0.007ab	1.67 ± 0.007ab	0.48 ± 0.009a	2.1 ± 0.004a	2.5 ± 0.007b	1.03 ± 0.004c	2.14 ± 0.004bc	2.18 ± 0.007ab
T8	2.02 ± 0.015a	1.87 ± 0.007ab	0.83 ± 0.005bc	0.36 ± 0.012a	1.2 ± 0.003b	1.2 ± 0.005c	0.92 ± 0.011c	2.19 ± 0.008b	2.21 ± 0.007a
T9	2.06 ± 0.007a	1.79 ± 0.008ab	1.89 ± 0.006a	0.39 ± 0.008a	1.9 ± 0.003a	1.4 ± 0.011c	1.11 ± 0.009b	2.40 ± 0.016a	2.25 ± 0.008a
T10	1.78 ± 0.007a	0.88 ± 0.006c	0.85 ± 0.005c	0.35 ± 0.007a	1.1 ± 0.011b	1.2 ± 0.005c	1.13 ± 0.012b	2.24 ± 0.007b	2.20 ± 0.008a
T11	1.62 ± 0.007a	0.92 ± 0.008c	0.75 ± 0.005c	0.37 ± 0.017a	1.8 ± 0.005a	1.5 ± 0.011c	1.04 ± 0.010c	2.26 ± 0.005b	2.25 ± 0.011a

Data are mean of three individual observation; ±1.0 = Standard error means of observed values; standard error followed by similar letter are not significantly different from each other; T1 = garden soil; T2 = garden soil + NPK (100%); T3 = garden soil + NPK (50%); T4 = garden soil + TP08; T5 = garden soil + TP16; T6 = garden soil + TP373; T7 = garden soil + consortia (TP06 + TP13 + TP14 + TP 27(7)); T8 = garden soil + TP08 + 50% NPK; T9 = garden soil + TP16 + 50% NPK; T10 = garden soil + TP373 + 50% NPK and T11 = garden soil + consortia (TP06 + TP13 + TP14 + TP 27(7)) + 50% NPK

On the other hand, the phosphatase and urease activity for both rice and yardlong bean cultivated soil was being increased in all the treatments as compared to control plant. The increment of phosphatase activity was 0.92–1.35 to 2.09–2.40 mg g^−1^ h^−1^ and the urease activity was found to be increased from 0.32–0.49 to 1.0–3.4 mg g^−1^ h^−1^. In case of the treatments only with NPK fertilizer (50 or 100% dose), only a slight increment was seen for both the phosphatase and urease activity. However, the increment was significantly higher (*p* ≤ 0.05) when treated with PSB alone. The lower enzyme activity in the NPK-treated soil may be contributed by the low population of phosphate solubilizers and nitrogen fixers. Results indicated that the application of agrochemicals significantly inhibits the population of phosphate solubilizers and nitrogen fixers, which was reported earlier by Balamurugan et al. (2010). It may be stated that the increase in phosphatase activity in PSB-treated soil is an indication of the increased soil fertility and improvement in the phosphate solubilization. Similarly, increase in urease activity in PSB-treated soil may be defined as the infrequent participation of PSBs in the nitrogen fixation. The efficiency of Phosphorus biofertilizers in biological nitrogen fixation was also reported by Kucey et al*.* (1989) and Gyaneshwar et al*.* (2002).

### Effect on NPK use efficiency

The NPK concentration, i.e., the availability was estimated in the soil of both rice and longyard bean at the first day and after 30th day of the treatments. Data presented in the Tables 3 and 4 showed that the NPK concentration in soil at the first day was 0.10 ± 0.001, 0.002 ± 0.0006 and 0.08 ± 0.002%, respectively, which was found to decreased up to 0.09 ± 0.002, 0.001 ± 0.0001 and 0.07 ± 0.002% due to uptake by the plants after 30 days. This higher increment over the control is due to the uptake by the plants in other treatments with PSB and NPK fertilizer as it was decreased up to a significantly (*p* ≤ 0.05) higher quantity.

**Table 3:** NPK use efficiency by the *Oryza sativa* (rice) grown in amendment of NPK fertilizers with the co-inoculation of PSB

Treatments	Nitrogen (N, %)			Phosphorus (P, %)			Potassium (K, %)		
	Availability in soil		Uptake by the leaves	Availability in soil		Uptake by the leaves	Availability in soil		Uptake by the leaves
	At 0 day	At 30th day	At 30th day	At 0 day	At 30th day	At 30th day	At 0 day	At 30th day	At 30th day
T1	0.10 ± 0.001c	0.09 ± 0.002c	3.13 ± 0.01d	0.002 ± 0.0006c	0.001 ± 0.0002c	0.021 ± 0.002d	0.08 ± 0.002c	0.07 ± 0.001d	1.82 ± 0.04d
T2	0.25 ± 0.002a	0.14 ± 0.001b	3.28 ± 0.06cd	0.007 ± 0.0003a	0.005 ± 0.0012a	0.030 ± 0.003c	0.19 ± 0.005a	0.19 ± 0.002a	2.91 ± 0.03c
T3	0.19 ± 0.005b	0.10 ± 0.001bc	3.44 ± 0.08cd	0.004 ± 0.0008b	0.003 ± 0.0022b	0.030 ± 0.002c	0.16 ± 0.008ab	0.11 ± 0.002bc	3.02 ± 0.02c
T4	0.18 ± 0.001b	0.12 ± 0.003b	5.25 ± 0.01a	0.002 ± 0.0009c	0.002 ± 0.0009bc	0.042 ± 0.001a	0.10 ± 0.011b	0.10 ± 0.011c	3.55 ± 0.09b
T5	0.20 ± 0.08b	0.13 ± 0.002b	3.90 ± 0.02c	0.002 ± 0.0010c	0.001 ± 0.0021c	0.041 ± 0.002a	0.12 ± 0.010b	0.10 ± 0.010c	3.79 ± 0.07ab
T6	0.22 ± 0.002ab	0.10 ± 0.002b	5.19 ± 0.07a	0.002 ± 0.0007c	0.002 ± 0.0011bc	0.041 ± 0.003a	0.12 ± 0.002b	0.09 ± 0.008c	3.88 ± 0.01ab
T7	0.21 ± 0.002b	0.15 ± 0.001b	4.26 ± 0.02b	0.002 ± 0.0002c	0.002 ± 0.0002bc	0.043 ± 0.002a	0.13 ± 0.003b	0.11 ± 0.002bc	3.68 ± 0.02b
T8	0.24 ± 0.001a	0.19 ± 0.006a	3.22 ± 0.03cd	0.004 ± 0.0005b	0.003 ± 0.0003b	0.034 ± 0.004b	0.23 ± 0.022a	0.13 ± 0.001b	4.08 ± 0.17a
T9	0.23 ± 0.004ab	0.22 ± 0.005a	3.82 ± 0.06c	0.004 ± 0.0003b	0.003 ± 0.0002b	0.037 ± 0.005ab	0.20 ± 0.008a	0.12 ± 0.001b	3.88 ± 0.02ab
T10	0.21 ± 0.002b	0.18 ± 0.002ab	3.73 ± 0.02c	0.004 ± 0.0002b	0.004 ± 0.0001a	0.036 ± 0.002b	0.19 ± 0.002a	0.14 ± 0.004b	3.77 ± 0.06ab
T11	0.26 ± 0.003a	0.19 ± 0.007a	2.91 ± 0.07d	0.004 ± 0.0009b	0.004 ± 0.0002a	0.038 ± 0.0009ab	0.21 ± 0.003a	0.13 ± 0.003b	3.97 ± 0.23a

Data are mean of three individual observation at 30 days of treatment; ±0.1 = standard error means of observed values; standard error bars followed by similar letter are not significantly different from each other; T1 = garden soil; T2 = garden soil + NPK (100%); T3 = garden soil + NPK (50%); T4 = garden soil + TP08; T5 = garden soil + TP16; T6 = garden soil + TP373; T7 = garden soil + consortia (TP06 + TP13 + TP14 + TP27(7)); T8 = garden soil + TP08 + 50% NPK; T9 = garden soil + TP16 + 50% NPK; T10 = garden soil + TP373 + 50% NPK and T11 = garden soil + consortia (TP06 + TP13 + TP14 + TP27(7)) + 50% NPK

**Table 4:** NPK use efficiency by the *Vigna unguiculata* (bean) grown in amendment of NPK fertilizers with the co-inoculation of PSB

Treatments	Nitrogen (N, %)			Phosphorus (P, %)			Potassium (K, %)		
	Availability in soil		Uptake by the leaves	Availability in soil		Uptake by the leaves	Availability in soil		Uptake by the leaves
	At 0 day	At 30th day	At 30th day	At 0 day	At 30th day	At 30th day	At 0 day	At 30th day	At 30th day
T1	0.10 ± 0.001c	0.09 ± 0.001cd	3.22 ± 0.01c	0.002 ± 0.006c	0.002 ± 0.0012b	0.023 ± 0.006c	0.09 ± 0.002bc	0.07 ± 0.002c	2.28 ± 0.07f
T2	0.25 ± 0.002a	0.23 ± 0.005a	3.53 ± 0.05bc	0.007 ± 0.003a	0.005 ± 0.0023a	0.038 ± 0.006ab	0.20 ± 0.005a	0.17 ± 0.002a	2.73 ± 0.10e
T3	0.19 ± 0.005b	0.17 ± 0.001b	3.62 ± 0.03b	0.004 ± 0.008b	0.004 ± 0.0006a	0.039 ± 0.008a	0.16 ± 0.008b	0.11 ± 0.004bc	2.88 ± 0.12e
T4	0.18 ± 0.001b	0.16 ± 0.002b	3.98 ± 0.08a	0.002 ± 0.009c	0.002 ± 0.0008b	0.038 ± 0.002ab	0.10 ± 0.011bc	0.10 ± 0.007bc	4.06 ± 0.0b
T5	0.20 ± 0.08b	0.18 ± 0.001ab	3.46 ± 0.01bc	0.002 ± 0.010c	0.001 ± 0.0007bc	0.043 ± 0.002a	0.12 ± 0.010b	0.10 ± 0.010bc	4.20 ± 0.02ab
T6	0.22 ± 0.002ab	0.13 ± 0.003bc	3.91 ± 0.02a	0.002 ± 0.007c	0.001 ± 0.0001bc	0.030 ± 0.002b	0.12 ± 0.002b	0.11 ± 0.003bc	4.00 ± 0.07b
T7	0.21 ± 0.002b	0.12 ± 0.002bc	3.27 ± 0.07c	0.002 ± 0.002c	0.001 ± 0.0001bc	0.042 ± 0.003a	0.13 ± 0.003b	0.09 ± 0.003bc	4.72 ± 0.03a
T8	0.24 ± 0.001a	0.21 ± 0.002a	3.11 ± 0.02cd	0.004 ± 0.005b	0.002 ± 0.0017b	0.043 ± 0.005a	0.23 ± 0.022a	0.12 ± 0.001b	3.55 ± 0.05c
T9	0.23 ± 0.004ab	0.13 ± 0.003bc	3.72 ± 0.02b	0.004 ± 0.003b	0.004 ± 0.0010a	0.042 ± 0.001a	0.20 ± 0.008a	0.13 ± 0.002b	3.47 ± 0.08c
T10	0.21 ± 0.002b	0.14 ± 0.004bc	3.70 ± 0.01b	0.004 ± 0.002b	0.003 ± 0.0008ab	0.039 ± 0.002a	0.20 ± 0.002a	0.11 ± 0.006b	3.50 ± 0.14c
T11	0.26 ± 0.003a	0.20 ± 0.001a	3.91 ± 0.02a	0.004 ± 0.002b	0.004 ± 0.0013a	0.041 ± 0.003a	0.21 ± 0.003a	0.13 ± 0.001b	3.21 ± 0.11cd

Data are mean of three individual observation at 30 days of treatment; ±0.1 = standard error means of observed values; standard error bars followed by similar letter are not significantly different from each other; T1 = garden soil; T2 = garden soil + NPK (100%); T3 = garden soil + NPK (50%); T4 = garden soil + TP08; T5 = garden soil + TP16; T6 = garden soil + TP373; T7 = garden soil + consortia (TP06 + TP13 + TP14 + TP27(7)); T8 = garden soil + TP08 + 50% NPK; T9 = garden soil + TP16 + 50% NPK; T10 = garden soil + TP373 + 50% NPK and T11 = garden soil + consortia (TP06 + TP13 + TP14 + TP27(7)) + 50% NPK

Again the uptake of NPK was analyzed by estimating their concentration in the leaves. In the case of the treatments with PSB alone or in consortia of compatible strains viz., TP06, TP13, TP14 and TP 27(7); and with or without the amendment of NPK fertilizer, the higher concentration of NPK was recorded in the leaves of both rice and longyard bean. The NPK concentration in leaves of rice was recorded at 3.22–5.25, 0.036–0.043 and 0.10–0.23% respectively, which is significantly higher (*p* ≤ 0.05 to 0.01) over the control plant (3.13–3.22, 0.021–0.023% and 1.82–2.28%, respectively). The same increment was also recorded in the case of longyard bean also. After the application of biofertilizers (PSB) and chemical fertilizers (urea, murate of potash and superphosphate); the NPK concentrations of both leaves and the soil are recorded at an inclined amount. A relatively higher uptake of NPK can be predicted in the treatment of externally applied NPK with the co-inoculation of PSB. This may be due to the presence of PSB which helps in the solubilization and thereby the uptake of NPK with or without NPK amendment by the plants. Increasing the availability of nutrients for the plants uptake with the application of biofertilizer was also reported earlier by several workers (Glick 1995; Rodriguez and Fraga 1999; Vessey 2003).

It was also seen that there was little difference in the level of NPK uptake by the plants with the treatments containing NPK in 50 and 100% dose. Thus, it is seen that a reduced application of NPK with the application of PSB can give a better result in NPK use efficiency. This is because of the low uptake of the externally applied NPK fertilizers by the crop plants which was also reported by the researchers Tilman (1998), Gyaneshwar et al*.* (2002) and Kennedy et al. (2004). It was also reported that the excessive application of NPK or in their higher dose, salt toxicity may be occurred, which may be accounted for the low nutrient uptake and reduced growth (Tilak et al. 2005; Nahed and Aziz 2007).

### Effect on seed emergence

**Fig. 1:**
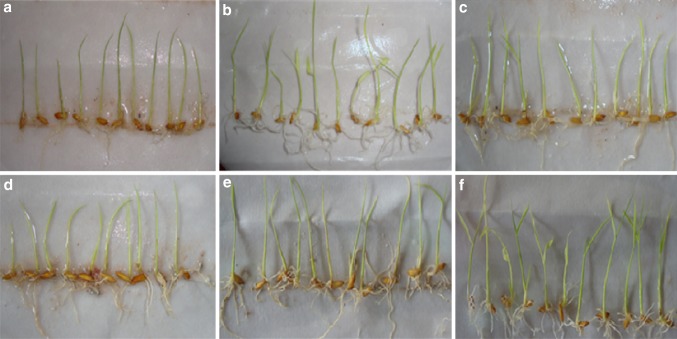
Effect of inoculation of PSB on germination of *Oryza sativa* grown under amendment of NPK fertilizer. **a** T1 (Control), **b** T2 (100% NPK), **c** T3 (50% NPK), **d** T6 (TP373), **e** T7 (Consortia of PSB), **f** T11 (Consortia of PSB + NPK)

Seed vigor is an important quality parameter to study the effect of PGPR in its germination. The effect of PSB on germination index is described in the Table 5 and Fig. 1. The seed having higher germination index (G. I.) is seen to be more vigorous than the others. A significantly higher G. I. (*p* ≤ 0.01) is obtained with the application of PSB alone or in the form of consortia of compatible strains, with or without external application of chemical NPK as compared to the control. Results have shown that the G. I., both in the case of rice and yardlong bean seed lot is increased; in rice seed from 2.50 ± 0.07 to 5.00 ± 0.10; and in yardlong bean seed it is increased from 2.70 ± 0.08 to 4.80 ± 0.07 through the co-inoculation of PSB with or without the external application of recommended dose of NPK fertilizer. It is seen that the treatment where PSB and NPK were treated, had a higher G. I. than the control as well as in NPK treated soil without PSB. Several workers also reported about the improvement of germination of different plants while growing through inoculation of PGPR (Vessey 2003; Poonguzhali et al. 2006).

**Table 5:** Effect of inoculation of phosphorous solubilizing bacteria on germination index of *Oryza sativa* and *Vigna unguiculata* grown in different concentration of NPK fertilizer at 5th day

Treatments	Germination index (G. I.)	
	*Oryza sativa*	V*igna unguiculata*
T1	2.50 ± 0.07c	2.70 ± 0.08c
T2	3.82 ± 0.12b	3.40 ± 0.11b
T3	3.87 ± 0.09b	3.39 ± 0.11b
T4	4.45 ± 0.13ab	3.50 ± 0.22b
T5	4.90 ± 0.08a	3.10 ± 0.10b
T6	4.37 ± 0.10ab	4.17 ± 0.09ab
T7	4.98 ± 0.07a	4.70 ± 0.12a
T8	4.45 ± 0.09b	3.45 ± 0.21b
T9	3.00 ± 0.11b	3.90 ± 0.06ab
T10	4.05 ± 0.05b	4.03 ± 0.11ab
T11	5.00 ± 0.10a	4.80 ± 0.07a

Data are mean of three individual observation; ±1.0 = Standard error means of observed values**;** standard error followed by similar letter are not significantly different from each other; T1 = garden soil; T2 = garden soil + NPK (100%); T3 = garden soil + NPK (50%); T4 = garden soil + TP08; T5 = garden soil + TP16; T6 = garden soil + TP373; T7 = garden soil + consortia (TP06 + TP13 + TP14 + TP 27(7)); T8 = garden soil + TP08 + 50% NPK; T9 = garden soil + TP16 + 50% NPK; T10 = garden soil + TP373 + 50% NPK and T11 = garden soil + consortia (TP06 + TP13 + TP14 + TP 27(7)) + 50% NPK

### Induction of amylase activity

**Fig. 2:**
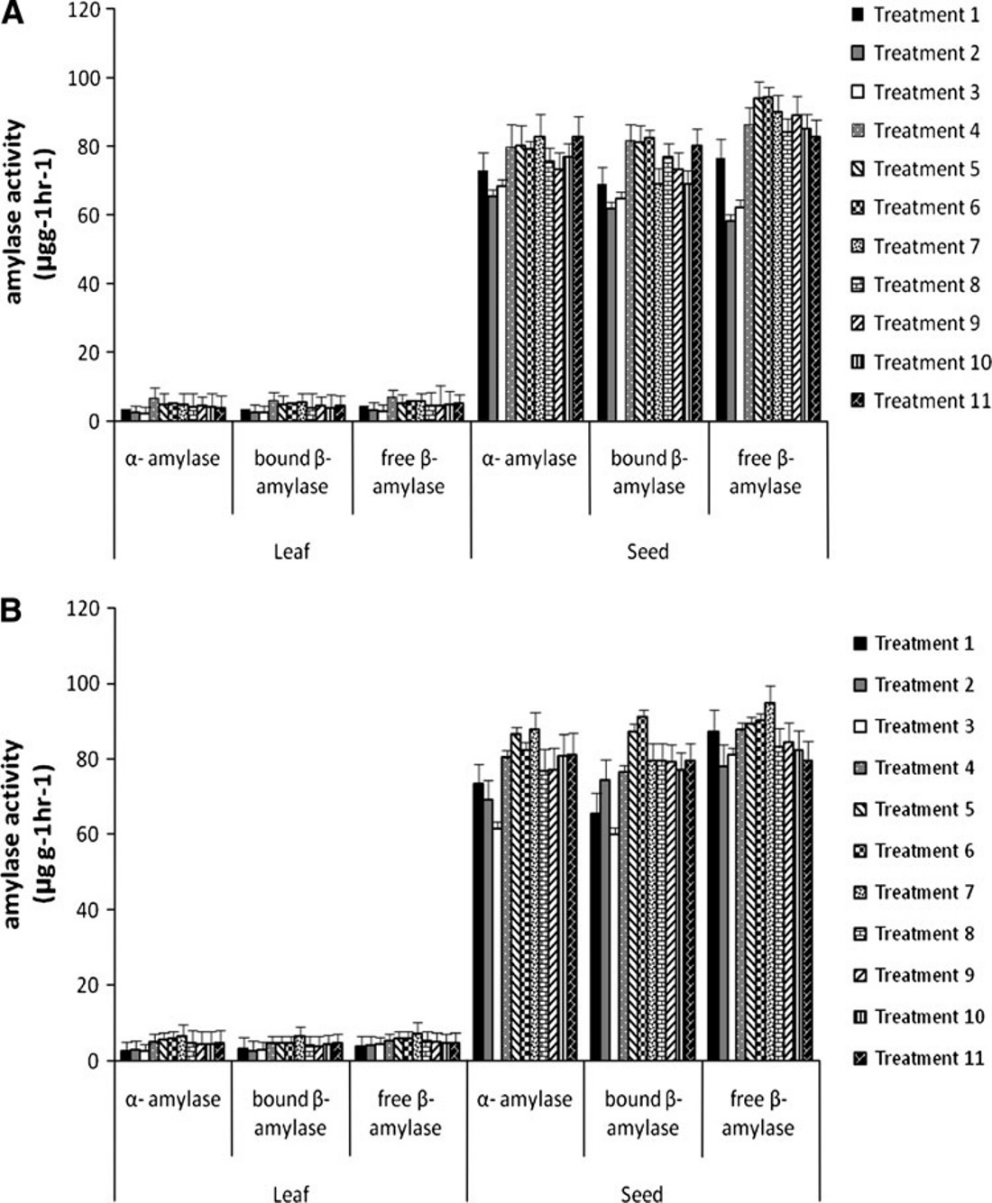
**a** Effect of inoculation of phosphorous solubilizing bacteria on amylase activity on *Oryza sativa* grown in amendment of NPK fertilizers. Data are mean of three individual observation; *error bars* standard error means of observed values; T1 = garden soil; T2 = garden soil + NPK (100%); T3 = garden soil + NPK (50%); T4 = garden soil + TP08; T5 = garden soil + TP16; T6 = garden soil + TP373; T7 = garden soil + consortia (TP06 + TP13 + TP14 + TP 27(7)); T8 = garden soil + TP08 + 50% NPK; T9 = garden soil + TP16 + 50% NPK; T10 = garden soil + TP373 + 50% NPK and T11 = garden soil + consortia (TP06 + TP13 + TP14 + TP 27(7)) + 50% NPK. **b** Effect of inoculation of phosphorous solubilizing bacteria on induction of amylase activity in *Vigna unguiculata* grown in amendment of NPK fertilizers. Data are mean of three individual observation; *error bars* standard error means of observed values; T1 = garden soil; T2 = garden soil + NPK (100%); T3 = garden soil + NPK (50%); T4 = garden soil + TP08; T5 = garden soil + TP16; T6 = garden soil + TP373; T7 = garden soil + consortia (TP06 + TP13 + TP14 + TP 27(7)); T8 = garden soil + TP08 + 50% NPK; T9 = garden soil + TP16 + 50% NPK; T10 = garden soil + TP373 + 50% NPK and T11 = garden soil + consortia (TP06 + TP13 + TP14 + TP 27(7)) + 50% NPK

The effect of NPK amendment and the co-inoculation of PSB on induction of amylase activity in rice and bean plant is shown in Fig. 2a and b. It was seen that the amylase activity of the treatments containing the PSB (T4–T11) was found to be significantly higher (*p* ≤ 0.05) than that of the control and the NPK alone (T2 and T3). It was also observed that the amylase activity in the seeds (58.4 ± 2.2−94.4 ± 3.0 μg g^−1^ h^−1^) were much higher (*p* ≤ 0.01) than that in the leaves which was recorded from 2.20 ± 1.1 to 6.5 ± 1.9 μg g^−1^ h^−1^. It is because of the more starch content in seed than the leaf which triggers the seed germination. During seed germination, amylase plays an important role in hydrolyzing the endosperm starch into sugars which provide energy for the growth of roots and shoots (Beck and Ziegler 1989; Kaneko et al. 2002). Therefore, it can be said that the induction of amylase activity during germination can be increased through the co-inoculation of PSB with or without the externally applied NPK fertilizer, which helps in seed germination and thereby the better growth of the plant.

### Effect on growth

**Fig. 3:**
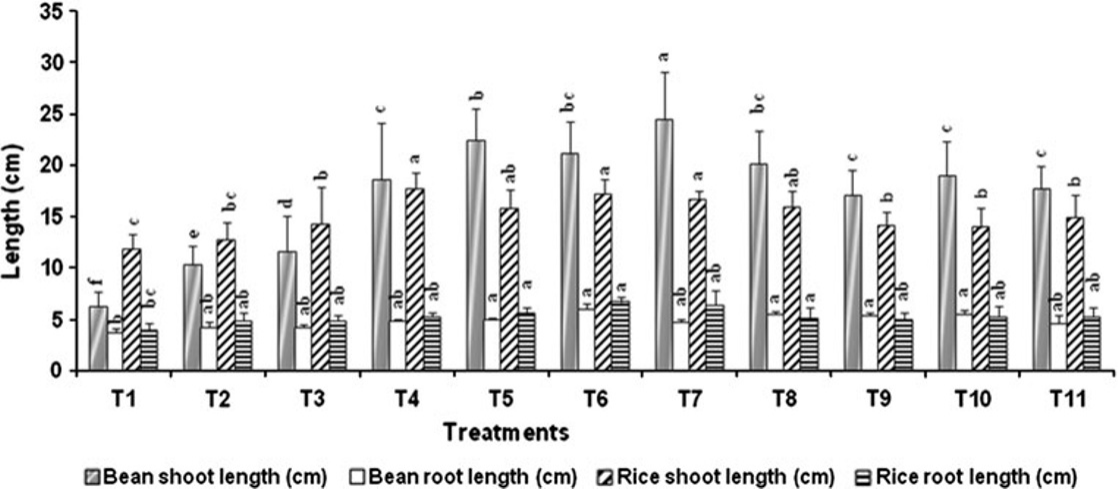
Comparison of effect of inoculation of phosphate solubilizing bacteria on root length–shooot length of *Oryza sativa* (rice) and *Vigna unguiculata* (longyard bean) grown in amendment of fertilizer. Data are mean of three individual observation;*error bars* standard error means of observed values; standard error bars followed by similar letter are not significantly different from each other T1 = garden soil; T2 = garden soil + NPK (100%); T3 = garden soil + NPK (50%); T4 = garden soil + TP08; T5 = garden soil + TP16; T6 = garden soil + TP373; T7 = garden soil + consortia (TP06 + TP13 + TP14 + TP 27(7)); T8 = garden soil + TP08 + 50% NPK; T9 = garden soil + TP16 + 50% NPK; T10 = garden soil + TP373 + 50% NPK and T11 = garden soil + consortia (TP06 + TP13 + TP14 + TP 27(7)) + 50% NPK

**Fig. 4:**
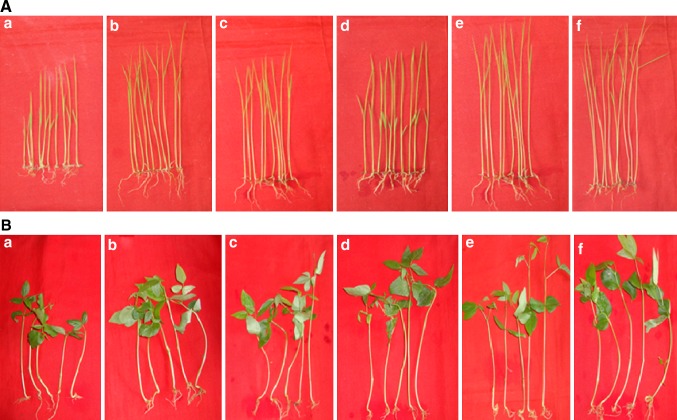
Comparison of the effect of co-inoculation of PSB on growth of *Oryza sativa* and *Vigna unguiculata* grown in amendment of NPK fertilizers. **A** Effect on growth of *Oryza sativa*. **B** Effect on growth of *Vigna unguiculata*. *a* T1 (Control); *b* T2 (100% NPK); *c* T3 (50% NPK); *d* T5 (PSB TP16); *e* T7 (Consortia of PSB, viz., TP06, TP13, TP14, TP 27(7)); *f* T11 (Consortia of PSB + NPK)

It is also seen that the root length, shoot length and the total biomass of the plants of each treatment containing the PSB with or without NPK fertilizer after 15th day (at V3 stage) was recorded and found to be significantly higher (*p* ≤ 0.05) than that of the control and the NPK alone (T2 and T3) (Figs. 3, 4a, b). In case of the treatments containing only PSB, i.e., without the NPK amendment, the changes were more. Due to the solubilization of phosphorus by PSB there may be higher uptake of NPK, which results in a better growth of the plant. In all the PSB treated yardlong bean*,* an appreciably higher amount of fresh weight and dry weight was recorded. The total enhancement recorded (data not shown) in case of fresh weight when compared to the control are ≥17 to >237% in case of roots and 0.5 to 71% in case of shoots. For dry weight the enhancements are ≥2.0 to >227% in case of roots and ≥5 to 157% in case of shoots. Highest root and shoot fresh weight was enhanced by the treatments TP373 and TP16; while in dry biomass for root and shoot was enhanced by the treatment through TP16 strain with 50% NPK and 100% NPK amendment.

On the other hand, there was no enhancement of root fresh weight in rice for the treatments T6, T8 and T9 and also showed lower root fresh weight for the treatment T10 and T11 as compared to the control. But in longyard bean, a relatively higher fresh weight (both for shoot and root) was found for all the treatments than the control (data not shown). Similar result was seen in the case for root dry weight also. From this observation it could be conclude that in 50% NPK, i. e., in reduced use of NPK and PSB treatment together achieved a higher growth than that of 100% NPK treatment alone. Thus, it can be predicted that the externally applied NPK fertilizer in excess amount or in recommended dose also is not may be beneficial for the crops. Similar use efficiency of NPK fertilizer by PSB and the utilization of PSB as biofertilizer in crop fields were earlier predicted by several workers (Rodriguez and Fraga 1999; Vessey 2003; Kennedy et al. 2004; Adesmoye and Kloepper 2009). Thus, use efficiency of NPK fertilizer can be improved through the application of PSB which helps in the growth of the plants through the uptake of NPK fertilizer.

## Conclusion

Low microbial populations followed by low enzyme activities have been evident with the application of excessive NPK fertilizers throughout this study. Our findings suggest that there is a strong relationship between fertilizer (NPK) and enzyme activity. Excessive use of NPK fertilizers leads to infertility of soil through a decreased level of microbial population with a subsequent decrease in dehydrogenase activity. Further, PSB applications increase the phosphatase and urease activities. Application of NPK fertilizers discourages this increment by reducing the population of PSB and nitrogen fixers. Moreover, the application of NPK in recommended dose or in its half dose gave the same results of soil enzyme activity, NPK uptake, germination and growth, with the introduction of PSB which implies reduced application of agrochemicals. Thus, the application of PSB in crop fields may be much beneficial for the agriculturist.

The uptake of NPK and plant growth promoting activity of PSB in both rice and yardlong bean can be explained by the occurrence of P solubilization as well as better scavenging of soluble P and other trace elements such as nitrogen, potassium, iron, zinc, etc. and also by production of plant growth promoting substances. Therefore, in support of the reduced application of NPK fertilizer and the high growth and yield of crops, the potential PSB strains can be employed as biofertilizing agents and to minimize the harmful effect of agrochemicals in the sustainable management of agriculture.
